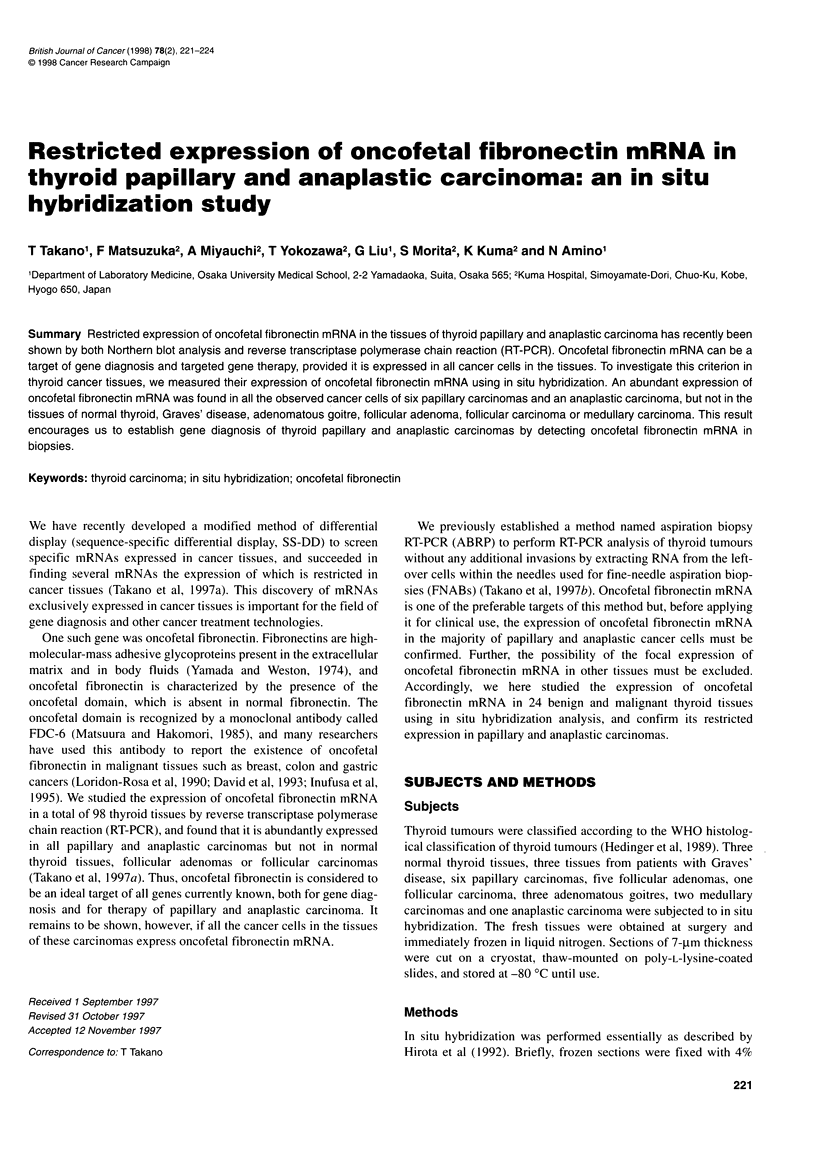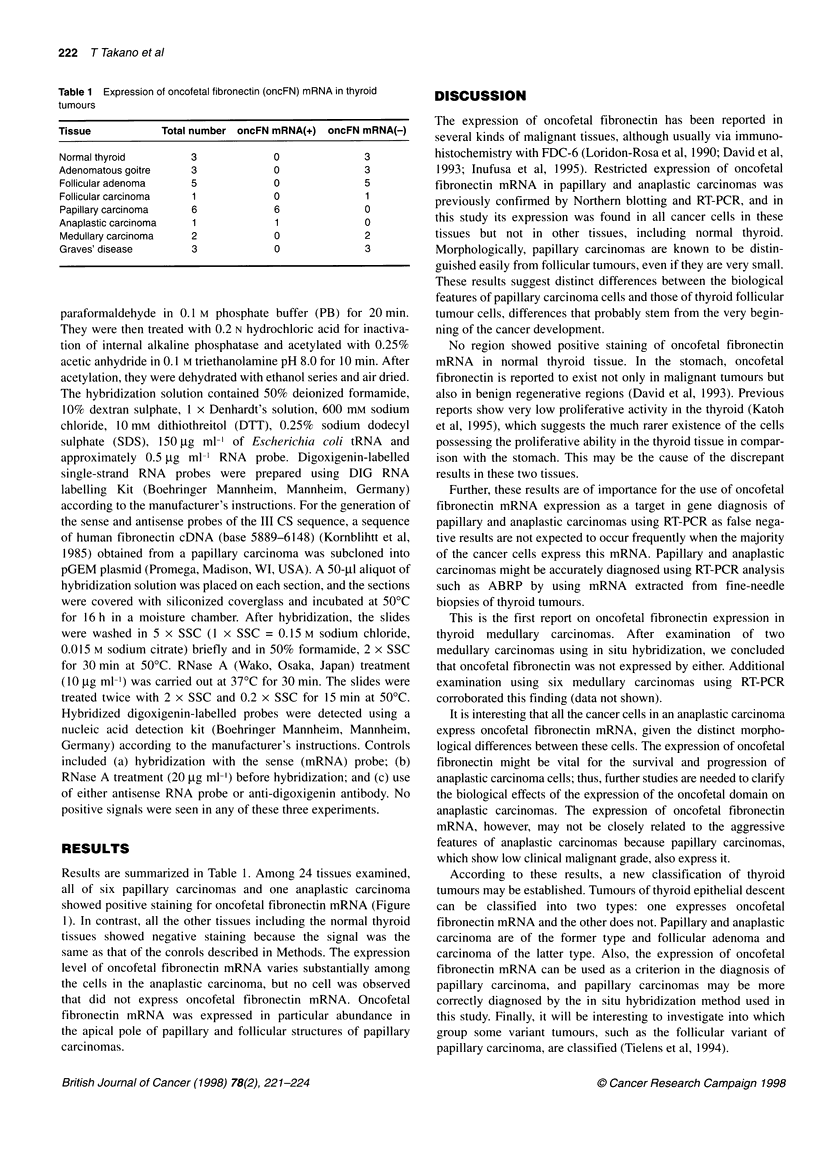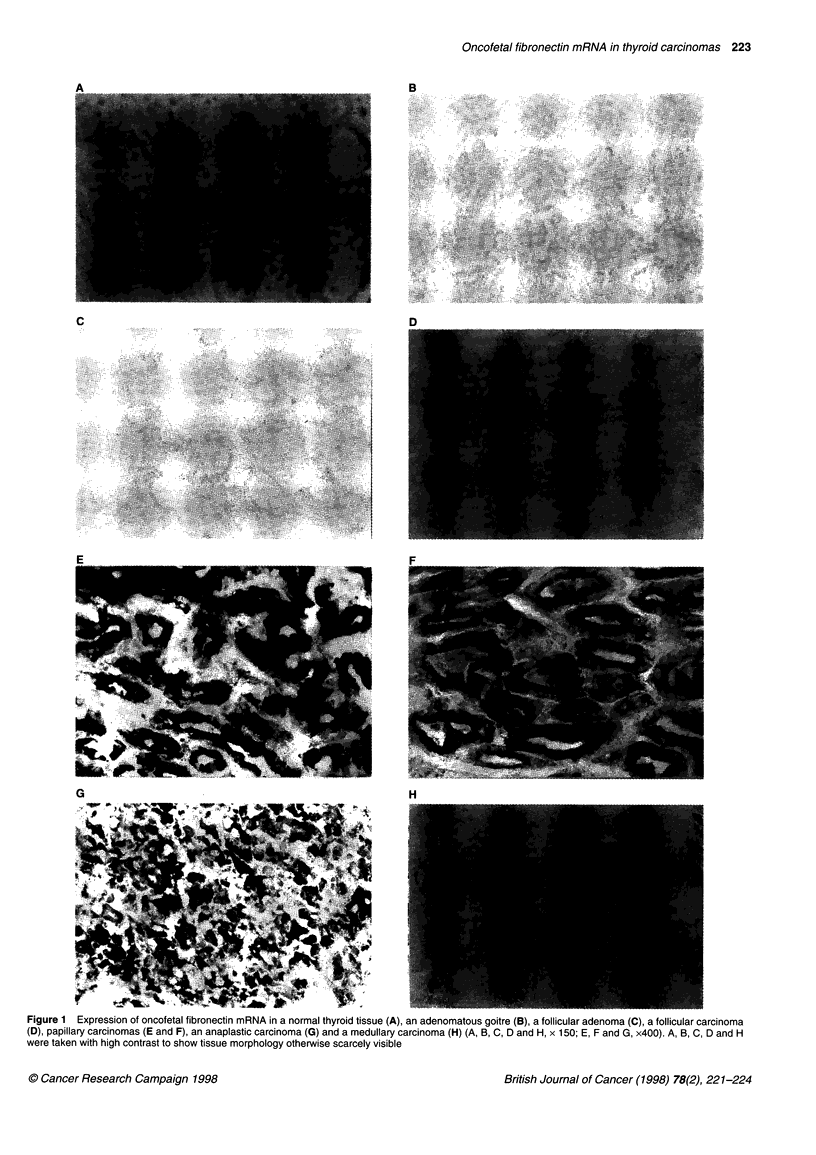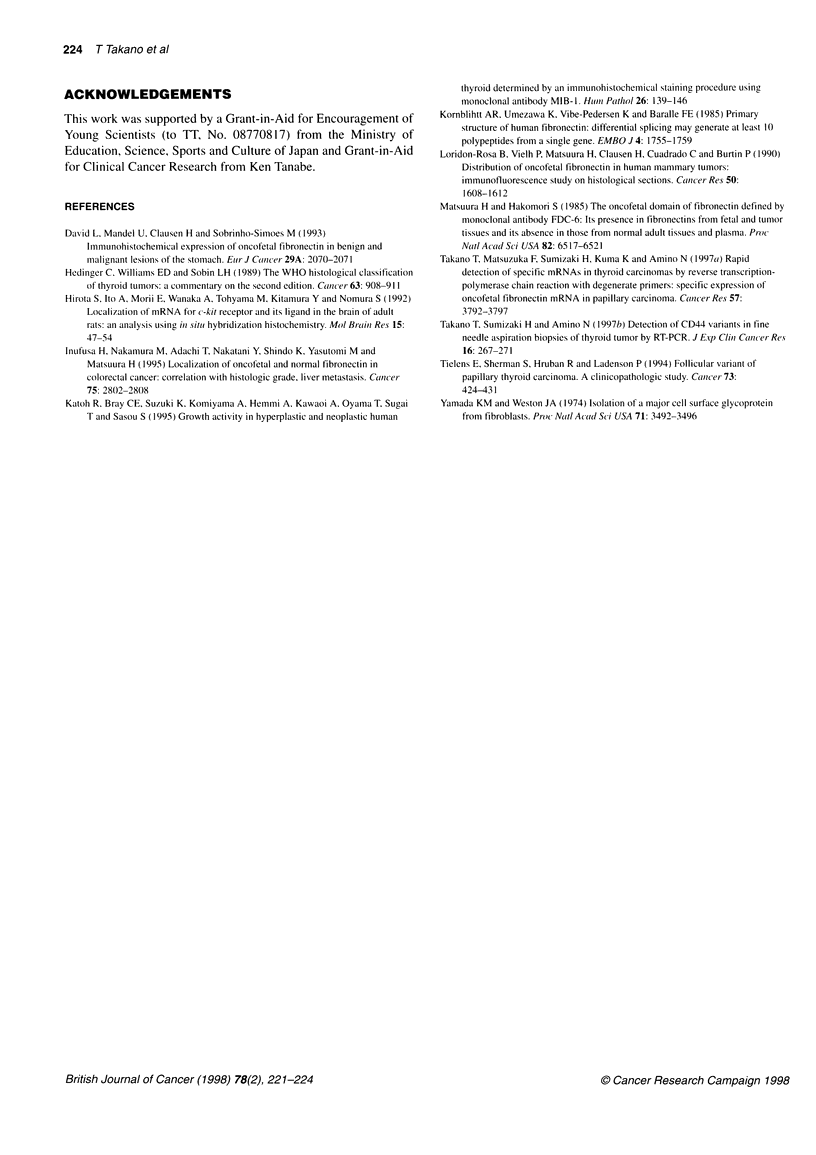# Restricted expression of oncofetal fibronectin mRNA in thyroid papillary and anaplastic carcinoma: an in situ hybridization study.

**DOI:** 10.1038/bjc.1998.468

**Published:** 1998-07

**Authors:** T. Takano, F. Matsuzuka, A. Miyauchi, T. Yokozawa, G. Liu, S. Morita, K. Kuma, N. Amino

**Affiliations:** Department of Laboratory Medicine, Osaka University Medical School, Suita, Japan.

## Abstract

**Images:**


British Journal of Cancer (1998) 78(2), 221–224
© 1998 Cancer Research Campaign

### Restricted expression of oncofetal fibronectin mRNA in thyroid papillary and anaplastic carcinoma: an in situ hybridization study

**T Takano[1], F Matsuzuka[2], A Miyauchi[2], T Yokozawa[2], G Liu[1], S Morita[2], K Kuma[2] and N Amino[1]**

[1]Department of Laboratory Medicine, Osaka University Medical School, 2-2 Yamadaoka, Suita, Osaka 565; [2]Kuma Hospital, Simoyamate-Dori, Chuo-Ku, Kobe, Hyogo 650, Japan

**Summary** Restricted expression of oncofetal fibronectin mRNA in the tissues of thyroid papillary and anaplastic carcinoma has recently been shown by both Northern blot analysis and reverse transcriptase polymerase chain reaction (RT-PCR). Oncofetal fibronectin mRNA can be a target of gene diagnosis and targeted gene therapy, provided it is expressed in all cancer cells in the tissues. To investigate this criterion in thyroid cancer tissues, we measured their expression of oncofetal fibronectin mRNA using in situ hybridization. An abundant expression of oncofetal fibronectin mRNA was found in all the observed cancer cells of six papillary carcinomas and an anaplastic carcinoma, but not in the tissues of normal thyroid, Graves' disease, adenomatous goitre, follicular adenoma, follicular carcinoma or medullary carcinoma. This result encourages us to establish gene diagnosis of thyroid papillary and anaplastic carcinomas by detecting oncofetal fibronectin mRNA in biopsies.

**Keywords:** thyroid carcinoma; in situ hybridization; oncofetal fibronectin

We have recently developed a modified method of differential display (sequence-specific differential display, SS-DD) to screen specific mRNAs expressed in cancer tissues, and succeeded in finding several mRNAs the expression of which is restricted in cancer tissues (Takano et al, 1997a). This discovery of mRNAs exclusively expressed in cancer tissues is important for the field of gene diagnosis and other cancer treatment technologies.

One such gene was oncofetal fibronectin. Fibronectins are high-molecular-mass adhesive glycoproteins present in the extracellular matrix and in body fluids (Yamada and Weston, 1974), and oncofetal fibronectin is characterized by the presence of the oncofetal domain, which is absent in normal fibronectin. The oncofetal domain is recognized by a monoclonal antibody called FDC-6 (Matsuura and Hakomori, 1985), and many researchers have used this antibody to report the existence of oncofetal fibronectin in malignant tissues such as breast, colon and gastric cancers (Loridon-Rosa et al, 1990; David et al, 1993; Inufusa et al, 1995). We studied the expression of oncofetal fibronectin mRNA in a total of 98 thyroid tissues by reverse transcriptase polymerase chain reaction (RT-PCR), and found that it is abundantly expressed in all papillary and anaplastic carcinomas but not in normal thyroid tissues, follicular adenomas or follicular carcinomas (Takano et al, 1997*a*). Thus, oncofetal fibronectin is considered to be an ideal target of all genes currently known, both for gene diagnosis and for therapy of papillary and anaplastic carcinoma. It remains to be shown, however, if all the cancer cells in the tissues of these carcinomas express oncofetal fibronectin mRNA.

*Received 1 September 1997*
*Revised 31 October 1997*
*Accepted 12 November 1997*

*Correspondence to:* T Takano

We previously established a method named aspiration biopsy RT-PCR (ABRP) to perform RT-PCR analysis of thyroid tumours without any additional invasions by extracting RNA from the left-over cells within the needles used for fine-needle aspiration biopsies (FNABs) (Takano et al, 1997*b*). Oncofetal fibronectin mRNA is one of the preferable targets of this method but, before applying it for clinical use, the expression of oncofetal fibronectin mRNA in the majority of papillary and anaplastic cancer cells must be confirmed. Further, the possibility of the focal expression of oncofetal fibronectin mRNA in other tissues must be excluded. Accordingly, we here studied the expression of oncofetal fibronectin mRNA in 24 benign and malignant thyroid tissues using in situ hybridization analysis, and confirm its restricted expression in papillary and anaplastic carcinomas.

### SUBJECTS AND METHODS

### Subjects

Thyroid tumours were classified according to the WHO histological classification of thyroid tumours (Hedinger et al, 1989). Three normal thyroid tissues, three tissues from patients with Graves' disease, six papillary carcinomas, five follicular adenomas, one follicular carcinoma, three adenomatous goitres, two medullary carcinomas and one anaplastic carcinoma were subjected to in situ hybridization. The fresh tissues were obtained at surgery and immediately frozen in liquid nitrogen. Sections of 7-μm thickness were cut on a cryostat, thaw-mounted on poly-L-lysine-coated slides, and stored at –80 °C until use.

### Methods

In situ hybridization was performed essentially as described by Hirota et al (1992). Briefly, frozen sections were fixed with 4%

**Table 1** Expression of oncofetal fibronectin (oncFN) mRNA in thyroid tumours

| Tissue | Total number | oncFN mRNA(+) | oncFN mRNA(–) |
|---|---|---|---|
| Normal thyroid | 3 | 0 | 3 |
| Adenomatous goitre | 3 | 0 | 3 |
| Follicular adenoma | 5 | 0 | 5 |
| Follicular carcinoma | 1 | 0 | 1 |
| Papillary carcinoma | 6 | 6 | 0 |
| Anaplastic carcinoma | 1 | 1 | 0 |
| Medullary carcinoma | 2 | 0 | 2 |
| Graves' disease | 3 | 0 | 3 |

paraformaldehyde in 0.1 M phosphate buffer (PB) for 20 min. They were then treated with 0.2 N hydrochloric acid for inactivation of internal alkaline phosphatase and acetylated with 0.25% acetic anhydride in 0.1 M triethanolamine pH 8.0 for 10 min. After acetylation, they were dehydrated with ethanol series and air dried. The hybridization solution contained 50% deionized formamide, 10% dextran sulphate, 1 × Denhardt's solution, 600 mM sodium chloride, 10 mM dithiothreitol (DTT), 0.25% sodium dodecyl sulphate (SDS), 150 μg $ml^{-1}$ of *Escherichia coli* tRNA and approximately 0.5 μg $ml^{-1}$ RNA probe. Digoxigenin-labelled single-strand RNA probes were prepared using DIG RNA labelling Kit (Boehringer Mannheim, Mannheim, Germany) according to the manufacturer's instructions. For the generation of the sense and antisense probes of the III CS sequence, a sequence of human fibronectin cDNA (base 5889–6148) (Kornblihtt et al, 1985) obtained from a papillary carcinoma was subcloned into pGEM plasmid (Promega, Madison, WI, USA). A 50-μl aliquot of hybridization solution was placed on each section, and the sections were covered with siliconized coverglass and incubated at 50°C for 16 h in a moisture chamber. After hybridization, the slides were washed in 5 × SSC (1 × SSC = 0.15 M sodium chloride, 0.015 M sodium citrate) briefly and in 50% formamide, 2 × SSC for 30 min at 50°C. RNase A (Wako, Osaka, Japan) treatment (10 μg $ml^{-1}$) was carried out at 37°C for 30 min. The slides were treated twice with 2 × SSC and 0.2 × SSC for 15 min at 50°C. Hybridized digoxigenin-labelled probes were detected using a nucleic acid detection kit (Boehringer Mannheim, Mannheim, Germany) according to the manufacturer's instructions. Controls included (a) hybridization with the sense (mRNA) probe; (b) RNase A treatment (20 μg $ml^{-1}$) before hybridization; and (c) use of either antisense RNA probe or anti-digoxigenin antibody. No positive signals were seen in any of these three experiments.

### RESULTS

Results are summarized in Table 1. Among 24 tissues examined, all of six papillary carcinomas and one anaplastic carcinoma showed positive staining for oncofetal fibronectin mRNA (Figure 1). In contrast, all the other tissues including the normal thyroid tissues showed negative staining because the signal was the same as that of the conrols described in Methods. The expression level of oncofetal fibronectin mRNA varies substantially among the cells in the anaplastic carcinoma, but no cell was observed that did not express oncofetal fibronectin mRNA. Oncofetal fibronectin mRNA was expressed in particular abundance in the apical pole of papillary and follicular structures of papillary carcinomas.

### DISCUSSION

The expression of oncofetal fibronectin has been reported in several kinds of malignant tissues, although usually via immunohistochemistry with FDC-6 (Loridon-Rosa et al, 1990; David et al, 1993; Inufusa et al, 1995). Restricted expression of oncofetal fibronectin mRNA in papillary and anaplastic carcinomas was previously confirmed by Northern blotting and RT-PCR, and in this study its expression was found in all cancer cells in these tissues but not in other tissues, including normal thyroid. Morphologically, papillary carcinomas are known to be distinguished easily from follicular tumours, even if they are very small. These results suggest distinct differences between the biological features of papillary carcinoma cells and those of thyroid follicular tumour cells, differences that probably stem from the very beginning of the cancer development.

No region showed positive staining of oncofetal fibronectin mRNA in normal thyroid tissue. In the stomach, oncofetal fibronectin is reported to exist not only in malignant tumours but also in benign regenerative regions (David et al, 1993). Previous reports show very low proliferative activity in the thyroid (Katoh et al, 1995), which suggests the much rarer existence of the cells possessing the proliferative ability in the thyroid tissue in comparison with the stomach. This may be the cause of the discrepant results in these two tissues.

Further, these results are of importance for the use of oncofetal fibronectin mRNA expression as a target in gene diagnosis of papillary and anaplastic carcinomas using RT-PCR as false negative results are not expected to occur frequently when the majority of the cancer cells express this mRNA. Papillary and anaplastic carcinomas might be accurately diagnosed using RT-PCR analysis such as ABRP by using mRNA extracted from fine-needle biopsies of thyroid tumours.

This is the first report on oncofetal fibronectin expression in thyroid medullary carcinomas. After examination of two medullary carcinomas using in situ hybridization, we concluded that oncofetal fibronectin was not expressed by either. Additional examination using six medullary carcinomas using RT-PCR corroborated this finding (data not shown).

It is interesting that all the cancer cells in an anaplastic carcinoma express oncofetal fibronectin mRNA, given the distinct morphological differences between these cells. The expression of oncofetal fibronectin might be vital for the survival and progression of anaplastic carcinoma cells; thus, further studies are needed to clarify the biological effects of the expression of the oncofetal domain on anaplastic carcinomas. The expression of oncofetal fibronectin mRNA, however, may not be closely related to the aggressive features of anaplastic carcinomas because papillary carcinomas, which show low clinical malignant grade, also express it.

According to these results, a new classification of thyroid tumours may be established. Tumours of thyroid epithelial descent can be classified into two types: one expresses oncofetal fibronectin mRNA and the other does not. Papillary and anaplastic carcinoma are of the former type and follicular adenoma and carcinoma of the latter type. Also, the expression of oncofetal fibronectin mRNA can be used as a criterion in the diagnosis of papillary carcinoma, and papillary carcinomas may be more correctly diagnosed by the in situ hybridization method used in this study. Finally, it will be interesting to investigate into which group some variant tumours, such as the follicular variant of papillary carcinoma, are classified (Tielens et al, 1994).

© Cancer Research Campaign 1998

**Figure 1** Expression of oncofetal fibronectin mRNA in a normal thyroid tissue (**A**), an adenomatous goitre (**B**), a follicular adenoma (**C**), a follicular carcinoma (**D**), papillary carcinomas (**E** and **F**), an anaplastic carcinoma (**G**) and a medullary carcinoma (**H**) (A, B, C, D and H, × 150; E, F and G, ×400). A, B, C, D and H were taken with high contrast to show tissue morphology otherwise scarcely visible

© Cancer Research Campaign 1998

### ACKNOWLEDGEMENTS

This work was supported by a Grant-in-Aid for Encouragement of Young Scientists (to TT, No. 08770817) from the Ministry of Education, Science, Sports and Culture of Japan and Grant-in-Aid for Clinical Cancer Research from Ken Tanabe.

### REFERENCES

David L, Mandel U, Clausen H and Sobrinho-Simoes M (1993) Immunohistochemical expression of oncofetal fibronectin in benign and malignant lesions of the stomach. *Eur J Cancer* **29A**: 2070–2071

Hedinger C, Williams ED and Sobin LH (1989) The WHO histological classification of thyroid tumors: a commentary on the second edition. *Cancer* **63**: 908–911

Hirota S, Ito A, Morii E, Wanaka A, Tohyama M, Kitamura Y and Nomura S (1992) Localization of mRNA for *c-kit* receptor and its ligand in the brain of adult rats: an analysis using *in situ* hybridization histochemistry. *Mol Brain Res* **15**: 47–54

Inufusa H, Nakamura M, Adachi T, Nakatani Y, Shindo K, Yasutomi M and Matsuura H (1995) Localization of oncofetal and normal fibronectin in colorectal cancer: correlation with histologic grade, liver metastasis. *Cancer* **75**: 2802–2808

Katoh R, Bray CE, Suzuki K, Komiyama A, Hemmi A, Kawaoi A, Oyama T, Sugai T and Sasou S (1995) Growth activity in hyperplastic and neoplastic human thyroid determined by an immunohistochemical staining procedure using monoclonal antibody MIB-1. *Hum Pathol* **26**: 139–146

Kornblihtt AR, Umezawa K, Vibe-Pedersen K and Baralle FE (1985) Primary structure of human fibronectin: differential splicing may generate at least 10 polypeptides from a single gene. *EMBO J* **4**: 1755–1759

Loridon-Rosa B, Vielh P, Matsuura H, Clausen H, Cuadrado C and Burtin P (1990) Distribution of oncofetal fibronectin in human mammary tumors: immunofluorescence study on histological sections. *Cancer Res* **50**: 1608–1612

Matsuura H and Hakomori S (1985) The oncofetal domain of fibronectin defined by monoclonal antibody FDC-6: Its presence in fibronectins from fetal and tumor tissues and its absence in those from normal adult tissues and plasma. *Proc Natl Acad Sci USA* **82**: 6517–6521

Takano T, Matsuzuka F, Sumizaki H, Kuma K and Amino N (1997*a*) Rapid detection of specific mRNAs in thyroid carcinomas by reverse transcription-polymerase chain reaction with degenerate primers: specific expression of oncofetal fibronectin mRNA in papillary carcinoma. *Cancer Res* **57**: 3792–3797

Takano T, Sumizaki H and Amino N (1997*b*) Detection of CD44 variants in fine needle aspiration biopsies of thyroid tumor by RT-PCR. *J Exp Clin Cancer Res* **16**: 267–271

Tielens E, Sherman S, Hruban R and Ladenson P (1994) Follicular variant of papillary thyroid carcinoma. A clinicopathologic study. *Cancer* **73**: 424–431

Yamada KM and Weston JA (1974) Isolation of a major cell surface glycoprotein from fibroblasts. *Proc Natl Acad Sci USA* **71**: 3492–3496

© Cancer Research Campaign 1998